# A Universal Toughening and Energy‐Dissipating Strategy for Impact‐Resistant 3D‐Printed Composites

**DOI:** 10.1002/advs.202501450

**Published:** 2025-04-09

**Authors:** Xiang Hong, Peng Wang, Yu Ma, Weidong Yang, Junming Zhang, Zhongsen Zhang, Yan Li

**Affiliations:** ^1^ School of Aerospace Engineering and Applied Mechanics Tongji University Shanghai 200092 P. R. China; ^2^ Beijing Institute of Technology Chongqing Innovation Center Chongqing 401120 P. R. China; ^3^ State Key Laboratory of Explosion Science and Technology Beijing Institute of Technology Beijing 100081 P. R. China; ^4^ Key Laboratory of AI‐aided Airworthiness of Civil Aircraft Structures Civil Aviation Administration Tongji University Shanghai 200092 P. R. China; ^5^ Shanghai Institute of Aircraft Mechanics and Control Shanghai 20092 P. R. China

**Keywords:** 3D‐printed composites, ductility, impact‐resistant, strength‐ductility synergy

## Abstract

3D‐printed polymer‐based composites are promising for various engineering applications due to high strength‐to‐weight ratios and design flexibility. However, conventional matrix materials, such as polylactic acid and epoxy resin, often exhibit brittleness and limited impact resistance (< 10 kJ m^−^^2^). Herein, a universal strategy is reported for enhancing the ductility and impact energy absorption of 3D‐printed composites by leveraging the dynamic crosslinking of B─O dative bonds. To validate its effectiveness, a smart composite (PLA/SSG) comprising shear‐stiffening gel fillers embedded in a polylactic acid matrix is designed and its rate‐dependent mechanical adjustability along with 3D printability is evaluated. The resulting composite shows significant improvements in impact resistance, ductility, and strength‐ductility balance. Specifically, the multiple crack and localized plastic yielding of polylactic acid matrix induced by shear‐stiffening gel fillers enables PLA/SSG with a 40‐times increase in ductility; the “soft‐hard” phase transition of shear‐stiffening gel induced by B─O bonds endows PLA/SSG with a 330% improvement in impact energy absorption. This B─O bonds‐inspired strategy provides a universal approach for printing smart impact‐resistant composites and structures.

## Graphical abstract

A universal toughening and energy dissipation strategy inspired by B─O bonds, which can significantly enhance the mechanical properties of composites, enables 3D‐printed smart impact‐resistant structures.

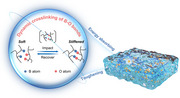

## 1 Introduction

3D‐printed polymer‐based composites offer many advantages such as lightweight, high strength, and design flexibility, making them widely used in aerospace and medical device applications.^[^ ^1^, ^2^, ^3^ ^]^ However, many polymers used in 3D printing are inherently brittle, such as thermoplastic polylactic acid PLA (which cannot undergo *β*‐transition at the molecular level^[^ ^4^ ^]^), thermosetting epoxy resins EP (whose 3D crosslinked network structure limits chain mobility^[^ ^5^ ^]^), and high molecular weight polystyrene polymers (which lacks flexible segments in its molecular chains^[^ ^6^ ^]^). This results in limited elongation at break (typically ε  <  10%) and impact strength (*E*  <  10 kJ m^−^^2^),^[^ ^4^, ^5^, ^6^ ^]^ particularly when notches, scratches, or internal defects are present. Therefore, it is essential to toughen these materials^[^ ^7^, ^8^, ^9^ ^]^ to expand the applications of 3D‐printed composites in high‐strain and high‐energy dissipating fields (ε > 30% and *E* > 30 kJ m^−^^2^).^[^ ^10^ ^]^

The toughening and energy dissipation strategies for polymer‐based composites primarily involve three approaches.^[^ ^10^, ^11^, ^12^ ^]^: i) flexible polymer addition, ii) rigid particle incorporation, and iii) reactive toughening. Among these, the flexible polymers (such as rubber, polyurethane, and polyamide) toughening strategy^[^ ^13^ ^]^ is the most common. The modulus of flexible polymers is typically lower than that of brittle matrices. This disparity can lead to local plastic yielding, absorbing energy and thereby enhancing the composite's ductility. While universal, it inevitably compromises mechanical integrity (modulus/strength reductions). For example, Chen et al.^[^ ^14^ ^]^ demonstrated this compromise through polyurethane‐toughened PLA with 225% ductility enhancement but ≈40% strength reduction. Similarly, nano‐rubber modified EP in Dong et al.^[^ ^15^ ^]^ achieved 3 times higher tensile ductility while retaining only 50% strength and 58% modulus versus unmodified EP. In contrast, incorporating rigid particles (such as nano‐SiO_2_/TiO_2_, graphene sheets, and nano‐clay)^[^ ^16^ ^]^ can suppress crack propagation and enhance the impact resistance, but shows less effect on ductility than flexible polymer addition. For example, Zhu et al.^[^ ^17^ ^]^ reported a micro‐epoxy/PLA composite with 2.2 times impact energy absorption yet fracture elongation was only 19.5%. Goyat et al.^[^ ^18^ ^]^ achieved 54% impact toughness enhancement in nano‐TiO_2_/EP with merely 18% ductility improvement. To overcome these limitations, researchers have proposed reactive toughening strategies (such as grafting reactions or copolymer modifications),^[^ ^19^ ^]^ aimed at improving the interfacial compatibility of constituents to achieve a balance between tensile strength, ductility, and impact resistance. For example, Battegazzore et al.^[^ ^20^ ^]^ demonstrated 100% tensile strength and 78% ductility gains in PHBH via epoxy coupling agents. Wang et al.^[^ ^21^ ^]^ used multifunctional epoxy oligomers as active compatibilizers, achieving 75.3 and 12.3 times improvements in both PLA's fracture elongation and impact resistance. However, this approach requires specific polymer compatibility, complex processing, and precise modifier dosage/time control.^[^ ^22^ ^]^ Therefore, it is imperative to develop a more universal, convenient, and effective toughening and energy dissipation strategy for 3D‐printed composites.

The B─O bond is a dative bond, characterized by strong bond strength (B─O bond ≈774.04 kJ mol^−1^ *vs*. C─C bond ≈334.72 kJ mol^−1^) and high thermodynamic stability.^[^ ^23^ ^]^ Its dynamic crosslinking properties enable more than 5 times energy dissipation.^[^ ^24^ ^]^ Based on Lewis acid‐base theory,^[^ ^23^ ^]^ B─O bonds can be strategically incorporated into polymer networks to synthesize polyborosiloxane‐based shear stiffening gel (SSG).^[^ ^25^ ^]^ At low strain rates, SSG exhibits good flexibility. While under high shear strain, it undergoes a “soft‐to‐hard” phase transition, leading to a rapid increase in the storage modulus by several orders of magnitude, demonstrating significant stiffness improvement.^[^ ^26^, ^27^, ^28^, ^29^ ^]^ In this context, incorporating SSG as a secondary filler into brittle polymer matrices offers a novel strategy to enhance the toughness and impact energy absorption of 3D‐printed composites. First, SSG can intelligently switch between “soft” and “hard” states under varying strain rates, absorbing impact energy and enabling the synergy between flexible and rigid particle toughening. Additionally, SSG exhibits a significantly temperature‐dependent viscosity (*η* = 4.94 × 10^4^ Pa·s at 25 °C to *η* = 5.86 × 10^3^ Pa·s at 100 °C)^[^ ^30^ ^]^ and excellent 3D printability,^[^ ^31^ ^]^ with minimal impact on the quality of the 3D‐printed composites when used in appropriate amounts. More importantly, the mobility mismatch between the flexible chains of SSG and the rigid polymer matrices suppresses chemical crosslinking,^[^ ^32^ ^]^ enabling a toughening strategy based on physical assembly with broad compatibility.

In this study, we propose a universal strategy for the toughening and energy‐dissipation of 3D‐printed composites based on the dynamic crosslinking of B─O dative bond. We apply this strategy to PLA, resulting in a smart anti‐impact composite (PLA/SSG) with a mechanically adjustable nature. At low strain rates, it achieves an excellent balance between ultimate strength and ductility; while at high strain rates, it demonstrates outstanding impact energy absorption. The strain rate‐induced “soft‐to‐hard” phase transition of SSG significantly enhanced the impact resistance, ductility, and strength‐ductility balance of PLA/SSG. Finally, we investigated the mechanical properties of PLA/SSG‐based fiber‐reinforced composites and demonstrated their promising application in the design of complex impact‐resistant structures through 3D printing.

## 2 Results and Discussion

### 2.1 Toughening and Energy‐Dissipating Strategy

**Figure 1:**
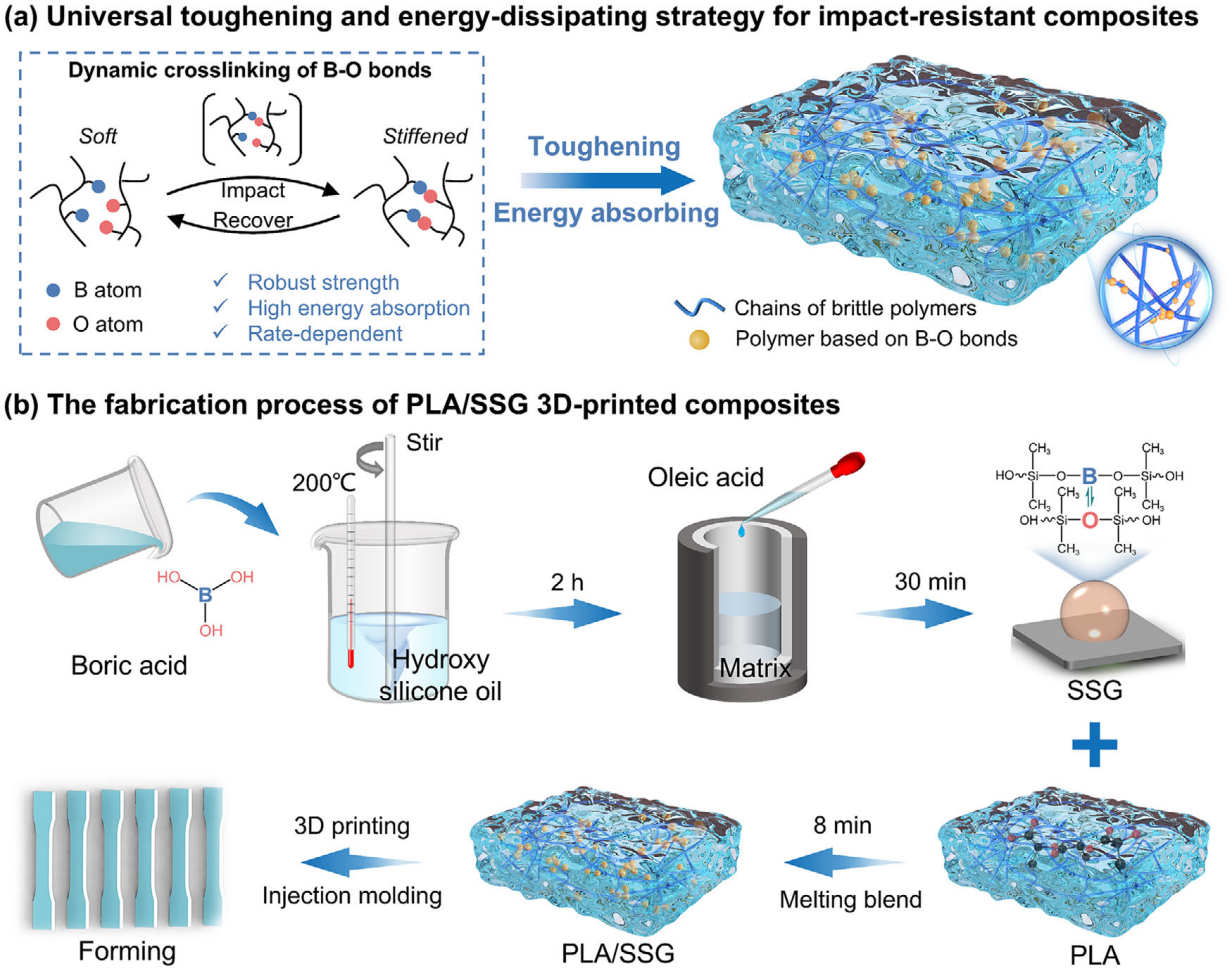
A universal strategy for ductile and impact‐resistant composites. a) A polymer based on B─O dynamic crosslinking bonds is introduced as a secondary filler into the brittle polymer matrix to enhance the toughness and impact energy absorption performance of 3D printed composites. b) Schematic illustration of the fabrication process of PLA/SSG, which includes the preparation of B─O polymer SSG, melt blending of PLA with SSG, and forming of PLA/SSG.

The electron deficiency of boron and electron richness of oxygen lead to the formation of B─O dative bond.^[^ ^24^ ^]^ In the natural state, B─O bonds are in a dynamic equilibrium of fracture and reorganization, and polymers based on B─O bonds are viscous. While under impact loading, rapid shear forces increase the reorganization rate of B─O bonds, forming more cross‐links and strengthening intermolecular forces, resulting in shear stiffening and a phase transition between the “soft” (viscous) and “hard” (glassy) state.^[^ ^23^, ^33^, ^34^ ^]^ We leverage this unique rate‐dependent behavior^[^ ^23^ ^]^ to develop a universal toughening and energy dissipation strategy for composites by embedding B─O containing polymers as secondary fillers within the 3D‐printed brittle polymer matrix (**Figure** **1**a).

To validate its effectiveness, we applied this strategy to PLA and resulted in PLA/SSG composites (Figure 1b). The B─O dynamic bonds were introduced via a dehydration condensation reaction. Under high temperatures, boric acid (H_3_BO_3_) reacts with the hydroxyl group (─OH) in hydroxyl‐terminated silicone oil (HO[(CH_3_)_2_SiO]_n_H) to eliminate water molecules, forming a polymer matrix with a B─O─Si crosslinked network. Moreover, a small amount of oleic acid is added to initiate a plasticization reaction, aiming to improve the processing performance and balance the dynamic response of the matrix. The resulting product (SSG) (Figure S1, Supporting Information) exhibits a soft state under slow compression, while it shows a rapid increase in stiffness under fast compression. Finally, the PLA/SSG (Figure S4, Supporting Information) was prepared by melt blending SSG filler at various weight percentages (Table S1, Supporting Information) with the PLA matrix.

### 2.2 Shear Stiffening Effect and Microstructure Characterization

**Figure 2:**
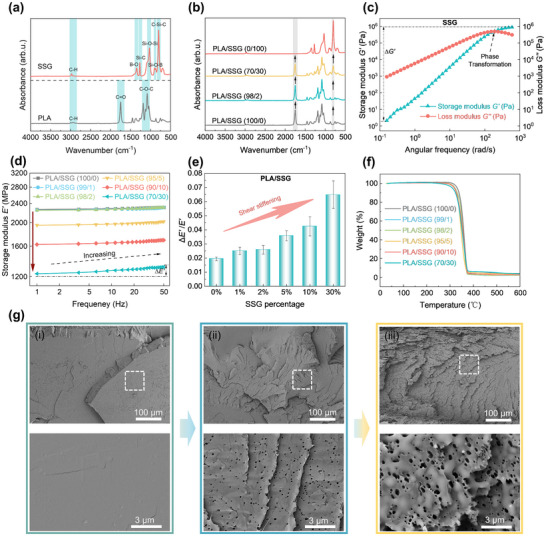
The shear stiffening effect and microstructure characterization of SSG and PLA/SSG. a,b) FTIR Spectra. c) The variation of SSG storage and loss modulus with shear frequency. The intersection of curves indicates the phase transition point between the liquid‐dominated state and the solid‐dominated state. d) The frequency‐dependent bending storage modulus of PLA/SSG. e) As the SSG percentage increased, the shear stiffening effect of PLA/SSG induced an increase in *∆E'/E'*. f) Weight loss percentage at different temperatures. g) Cross‐sectional morphology of PLA/SSG after freeze‐fracture with a typical phase‐separated "sea‐island" structure i). PLA/SSG (100/0), ii). PLA/SSG (98/2), iii). PLA/SSG (70/30)).

We characterized the chemical composition of SSG. Characteristic peaks at ≈1342 and 886 cm^−1^ (**Figure** **2**a) correspond to B─O, indicating condensation polymerization between the hydroxyl groups of silicone oil and boric acid.^[^ ^33^ ^]^ Strong absorption at 1020 cm^−1^ confirms that SSG is a polymer system with a Si─O─Si backbone (Figure S5, Supporting Information). The 1753 cm^−^¹ peak represents the carbonyl C═O stretching of PLA, and its intensity decreases as SSG content increases (Figure 2b), indicating the uniformity of melt blending. Besides, the absence of new peaks in the composite system (Figure 2b) indicates that the addition of SSG results in a physical mixture, ensuring compatibility with common 3D printing polymers.

Next, we investigated the shear‐stiffening effect of materials. For SSG, at low angular frequencies, the loss modulus (*G″*) is significantly greater than its storage modulus (*G′*) (Figure 2c), indicating good flow properties. At this stage, the B─O bonds are in a dynamic equilibrium between fracture and reorganization. As the angular frequency increases, the rate of B─O bond reorganization gradually surpasses the rate of fracture, leading to a reduction in the difference between *G′* and *G″*, and to an increase in stiffness.^[^ ^24^ ^]^ Until 188 rad s^−1^, the molecular chain crosslinking density reaches the critical point, causing SSG to transition from a liquid‐dominated to a solid‐dominated state.^[^ ^33^ ^]^ For PLA/SSG, the bending storage modulus usually decreases with increasing SSG content (Figure 2d), which is primarily due to the flexibility of SSG.^[^ ^35^ ^]^ Further analyzing the storage modulus difference (Figure 2e), *∆E'/E'* rises from 0.019 to 0.065 as the SSG content rises from 0% to 30%, which can be attributed to the shear stiffening effect of the SSG phase. Additionally, the initial decomposition temperature of all composites remains ≈280°C (Figure 2f), indicating that PLA/SSG retains good thermal stability.

Figure 2g[i] shows the cross‐sectional morphology of PLA, which is smooth and flat, exhibiting brittle fracture characteristics. In contrast, PLA/SSG exhibits ductile fracture (Figures 2g[ii] and [iii]), where the SSG phase is uniformly dispersed within the PLA matrix, forming a “sea‐island” structure. This dispersion acts as a stress concentrator,^[^ ^36^ ^]^ inducing plastic yielding of the matrix and resulting in a rough fracture surface with dense fine river patterns. In addition, due to the small initial particle size of the SSG, its higher specific surface area and surface energy keep it in an unstable energy state.^[^ ^37^ ^]^ As the weight percentage increases, the SSG particles tend to aggregate, resulting in an increase in average particle size from 470 to 697 nm (Figures S6 and S7, Supporting Information).

### 2.3 Strength‐Ductility Balance and Impact‐Resistance

**Figure 3:**
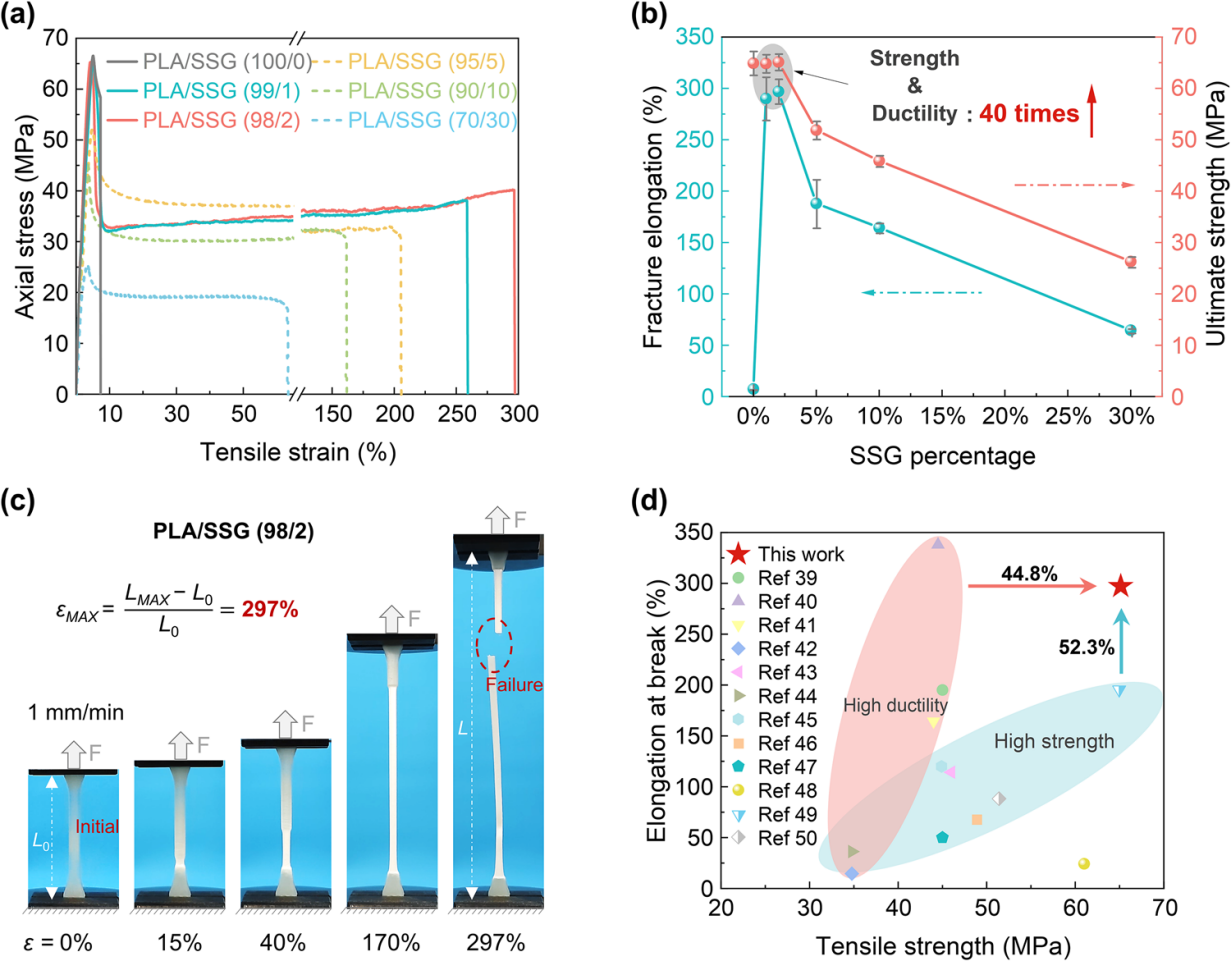
Strength‐ductility balance at low strain rates. a) Quasi‐static tensile stress‐strain curve. b) Elongation at tensile failure and ultimate strength of different types of PLA/SSG. At SSG% = 2%, PLA/SSG exhibits a maximum fracture strain of 40 times. c) Significant necking deformation of PLA/SSG before fracture. d) Exhibiting a balance between strength and ductility,^[^ ^39^, ^40^, ^41^, ^42^, ^43^, ^44^, ^45^, ^46^, ^47^, ^48^, ^49^, ^50^ ^]^ with a 44.8% increase in ultimate tensile strength and a 52.3% increase in ductility.

To characterize the strength and ductility of PLA/SSG at low strain rates, we conducted quasi‐static tensile tests. PLA exhibited limited deformability and a typical brittle fracture pattern under tensile loading, with a maximum fracture strain of only 7%. In contrast, PLA/SSG demonstrated significantly enhanced ductility, achieving a maximum fracture strain of 297% (**Figures** **3**a; Figure S8, Supporting Information). This improvement is evidenced by the substantial necking deformation observed before fracture (Figure 3c; Figure S9, Supporting Information).

As SSG content increases from 0% to 30%, the fracture strain initially increases to 297% and then gradually decreases to 65%, while the ultimate tensile strength always declines (Figure 3b). Notably, when SSG content is ≈1 ≈2 wt.%, PLA/SSG exhibits the optimal balance of strength and ductility, with the fracture strain increased more than 40 times. This can be attributed to the deformation of SSG, alongside the micro‐scale multiple cracking and shear‐yielding mechanisms of PLA.^[^ ^25^, ^38^ ^]^ Specifically, multiple cracks interact and hinder each other, preventing destructive fracture. Besides, the stress field at the tips of cracks will induce shear yielding in the PLA matrix, forming a highly oriented shear band at ≈45° (Figures S12, Supporting Information), leading to an increase in ductility. Compared to previous studies,^[^ ^39^, ^40^, ^41^, ^42^, ^43^, ^44^, ^45^, ^46^, ^47^, ^48^, ^49^, ^50^ ^]^ our composites demonstrate improvements of 44.8% in ultimate tensile strength and 52.3% in ductility (Figure 3d).

**Figure 4:**
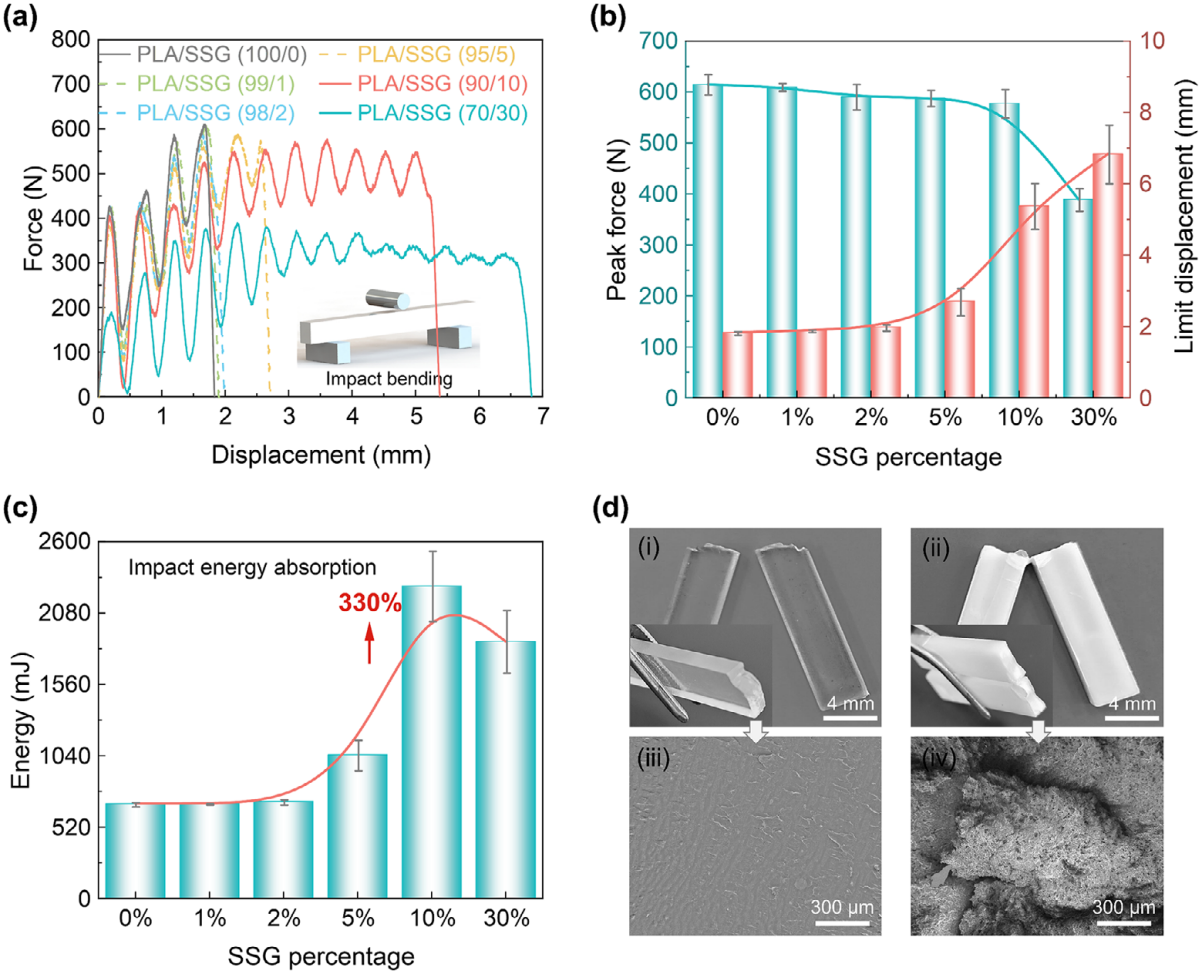
Impact resistance at high strain rates. a) Impact bending load‐displacement curve. b) Impact peak load and maximum displacement of different types of PLA/SSG. c) Comparison of impact energy absorption. d) The smooth brittle fracture surfaces of PLA (i and iii), and the rough ductile fracture surfaces of PLA/SSG (ii and iv).

To evaluate the energy absorption properties of PLA/SSG at high strain rates, we conducted impact bending tests^[^ ^51^ ^]^ (**Figure** **4**a). PLA/SSG exhibits lower peak force and a longer load plateau. Moreover, with the increase of SSG content (Figure 4b), the peak force of PLA/SSG decreases from 615 to 389 N, while the maximum displacement rises from 1.8 to 6.9 mm, indicating superior cushioning performance and enhanced energy absorption.^[^ ^25^, ^52^ ^]^

Among all tested samples (Figure 4c), PLA/SSG (90/10) showed the highest impact energy absorption (2277 mJ), 330% higher than PLA, which can be attributed to the shear‐stiffening effect of SSG.^[^ ^34^ ^]^ Interestingly, the energy absorption of PLA/SSG did not continuously increase with higher SSG content. In fact, PLA/SSG (70/30) exhibited lower impact energy absorption than PLA/SSG (90/10). This unexpected behavior is primarily due to the larger particle size of the SSG microspheres in PLA/SSG (70/30) (Figure 2g), which induces pronounced stress concentration within the PLA matrix, resulting in rapid crack propagation both within the SSG phase and at the interface.^[^ ^17^ ^]^ We further compared the fracture modes of select samples. For PLA (Figures 4d[i] and [iii]), the fracture surface is quite smooth, showing limited plastic deformation and energy dissipation. The sample fractured completely, with notches appearing at the impact edges. In contrast, PLA/SSG (90/10) maintained a continuous structure after impact (Figures 4d[ii] and [iv]). Its fracture surfaces appeared much rougher, with numerous stress‐whitening regions^[^ ^11^ ^]^ (Figures S13, Supporting Information), exhibiting typical features of ductile fracture and significant energy dissipation.

### 2.4 Toughening and Energy Absorption Mechanism

**Figure 5:**
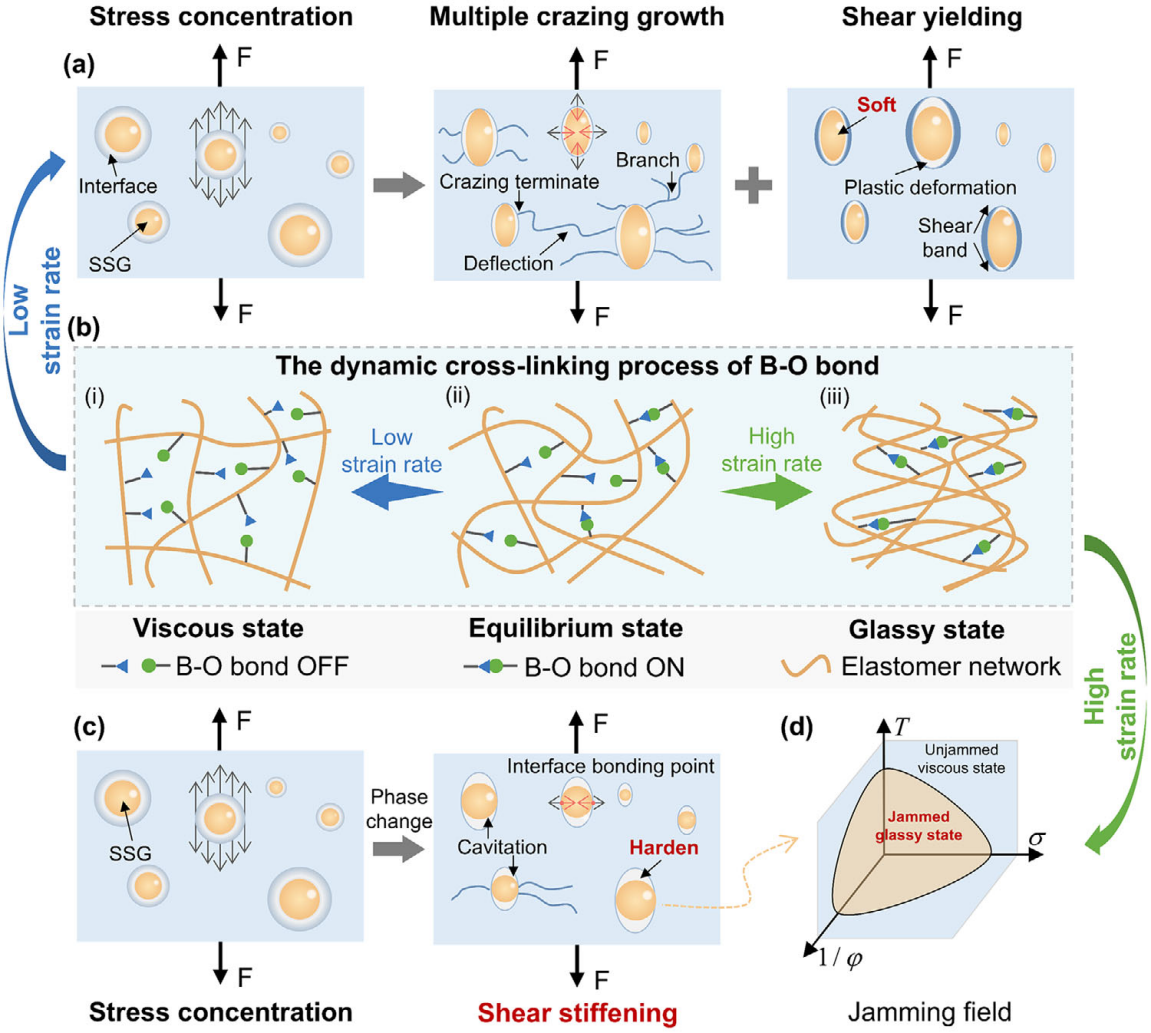
Toughening and energy absorption mechanisms. a) Toughening mechanisms of PLA/SSG at low strain rates (multiple crazing and shear yielding). b) Schematic diagram of molecular chain entanglement and disentanglement in SSG under different strain rates. c) Energy absorption mechanism of PLA/SSG at high strain rates (shear stiffening). d) The jamming phase diagram of SSG.

At low strain rates, the B─O dynamic bonds have sufficient time to break and reorganize, allowing the SSG molecular chains to easily disentangle (**Figure** **5**b[i]), and resulting in a viscoelastic solid state at the macroscopic level. In this state, the toughening mechanism of SSG for PLA is similar to that of flexible polymers, primarily involving multiple crazing and shear yielding.^[^ ^38^, ^39^, ^53^ ^]^ Specifically, due to the biphasic nature of the PLA/SSG, its internal stress field is non‐uniform. Under tensile loading, stress concentrates on the surface of the SSG microspheres, leading to a triaxial tensile stress field in the surrounding regions. On the one hand, the tensile stress field induces numerous microcracks within the PLA matrix, which either terminate or branch upon encountering SSG, as confirmed by Figure S14 (Supporting Information). This process prevents the formation of macroscopic cracks, thereby enhancing the ductility of composites. On the other hand, the tensile stress field induces interfacial debonding between PLA and SSG (Figure S14, Supporting Information), leading to cavitation and triggering plastic shear yielding in PLA, which further improves the ductility of PLA/SSG.

At high strain rates, the shear stiffening behavior of SSG absorbs impact energy.^[^ ^54^ ^]^ Specifically, when PLA/SSG is subjected to impact loading, the triaxial tensile stress field generated by stress concentration will rapidly act on the SSG microspheres. At this point, the time scale for disentanglement is much shorter than the characteristic time for B─O dynamic crosslinking,^[^ ^22^ ^]^ resulting in restricted molecular chain movement and entanglement (Figure 5b[iii]), which significantly increases the molecular chain packing fraction density φ of SSG. According to the jamming field theory,^[^ ^55^ ^]^ the onset of the “soft‐to‐hard” transition in polymers associated with jamming depends on the interplay between temperature *T*, applied stress σ, and inverse of the packing fraction density 1/φ (Figure 5d). The increased φ can jam the liquid‐like SSG,^[^ ^56^ ^]^ causing it to transition from the viscous state to a glassy state, resulting in a rapid increase in stiffness (Figure 2c) and absorbing kinetic energy.^[^ ^34^ ^]^ Furthermore, the jamming of molecular chains leads to a reduction in the volume of the SSG microspheres, causing cavitation and debonding at the interface (Figure S14, Supporting Information). The macromolecular chains of PLA undergo plastic flow, and the impact energy will be further dissipated through multiple cracks and shear yielding.

### 2.5 PLA/SSG‐Based Impact‐Resistant Structures

**Figure 6:**
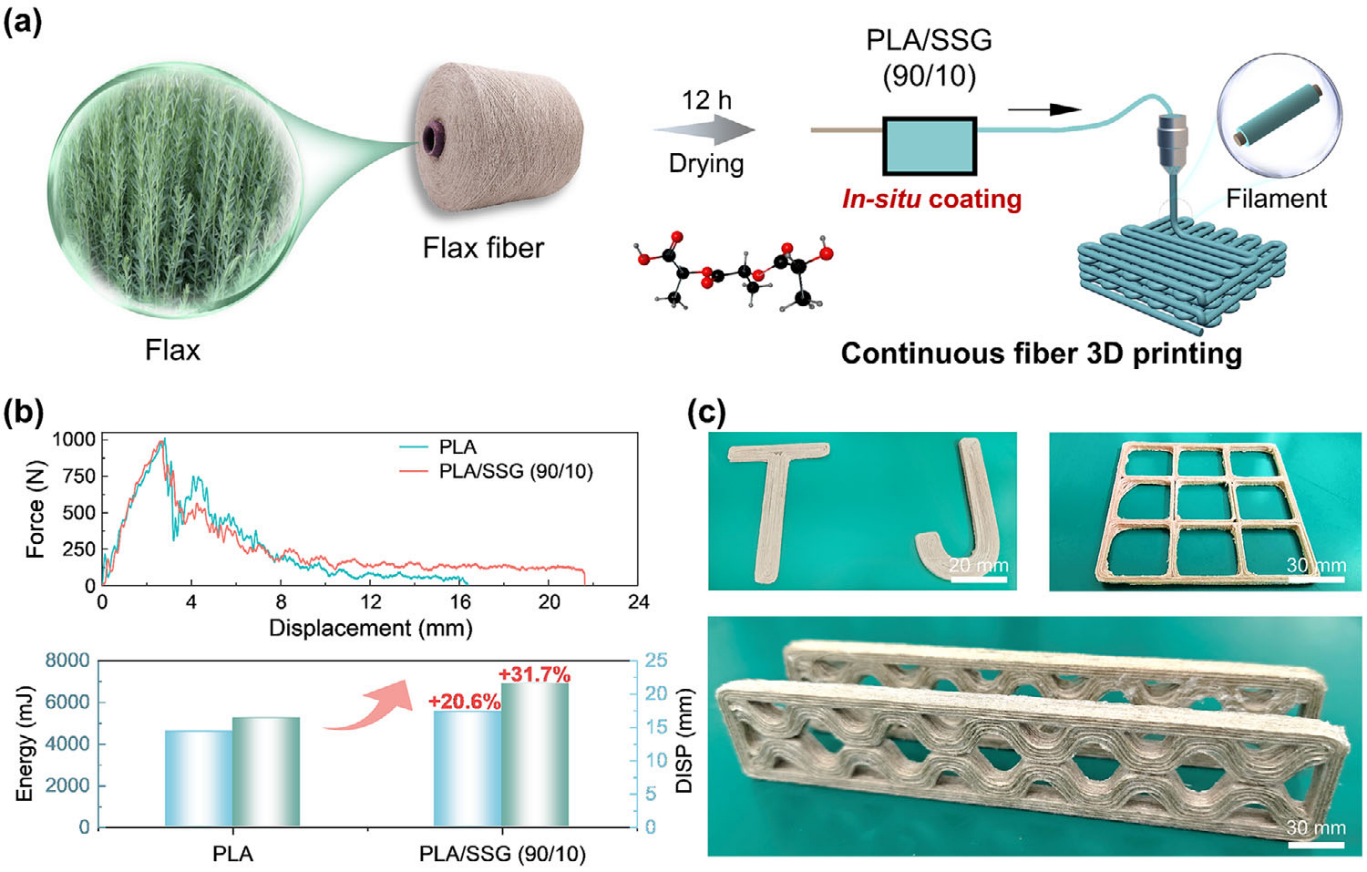
The PLA/SSG‐based impact‐resistant structures. a) The in situ coating process of prepreg filaments and the continuous fiber 3D printing workflow. b) The impact‐resistant performance of PLA/SSG‐based flax fiber reinforced composites. c) Different 3D printing structures.

The high ductility and impact energy absorption of PLA/SSG offers a unique solution to the inherent brittleness of 3D printed polymer composites. To demonstrate this, we employed the in situ coating process (Figure S15, Supporting Information) and continuous fiber 3D printing techniques (**Figure** **6**a) to fabricate flax fiber‐reinforced composite impact specimens (80 mm × 10 mm × 8 mm) and tested them with an impact energy of 30 J (Figure S10, Supporting Information). Compared to the traditional PLA‐based composites, the PLA/SSG‐based exhibited improvements of 31.7% in maximum impact displacement and 20.6% in energy absorption (Figure 6b), demonstrating a significant toughening effect. Additionally, taking advantage of the thermal stability and processability of PLA/SSG, we successfully fabricated various structures (Figure 6c). The printing process was smooth, and the formed parts displayed excellent quality, confirming the potential of PLA/SSG in the design of complex impact‐resistant structures.

## 3 Conclusion

This work presents a universal toughening and energy‐dissipation strategy for 3D‐printed composites, inspired by the dynamic crosslinking of the B─O dative bond. PLA/SSG is designed by embedding SSG fillers within a PLA matrix, enabling a mechanically adjustable nature. The composite exhibits remarkable enhancements in impact resistance, ductility, and strength‐ductility balance, making it particularly well‐suited for impact protection applications. Specifically, it achieves a 40‐times increase in ductility while preserving tensile strength, and a 330% improvement in impact energy absorption. Besides, SSG/PLA shows outstanding thermal stability and processability, allowing the customization of complex 3D‐printed structures. Leveraging these natures, a PLA/SSG‐based flax fiber‐reinforced composite is fabricated, achieving 20.6% higher energy dissipation than traditional composites. We believe that the universal strategy inspired by the B─O bond can be readily applied to other brittle polymers, providing a simple and effective method for developing 3D‐printed impact‐resistant composites and related structures.

## 4 Experimental Section

### Materials

Boric acid (H_3_BO_3_), hydroxyl silicone oil (HO[(CH_3_)_2_SiO]_n_H), and oleic acid (C_18_H_34_O_2_) were procured from Macklin Biochemical Co., Ltd., Shanghai, China. Polylactic acid (PLA‐4032D) was acquired from NatureWorks, America.

### The Preparation of SSG

First, boric acid and hydroxyl silicone oil were thoroughly mixed at a concentration of 80 mg mL^−1^. The mixture was subjected to a polymerization reaction at 200°C, stirring every 20 min for 2 h to obtain a polymer matrix with large amounts of silk‐like luster. Next, 1 wt.% of oleic acid was added to the matrix, and the mixture was continuously stirred for 30 min to undergo the plasticization reaction. Finally, after cooling to room temperature, the product (SSG) was obtained.

### The Preparation of PLA/SSG

The PLA/SSG composites were prepared using a melt blending process. The processing temperature was 200°C, with a screw speed of 60 rpm for 8 min. The mixing ratios are detailed in Table S1 (Supporting Information). To ensure uniform mixing, pre‐blending of PLA and SSG was necessary. To prevent moisture interference, both the raw materials and the PLA/SSG were dried before and after blending.

### The Preparation of Specimens

The standard tensile and impact test specimens for PLA/SSG were produced using an injection molding process. The barrel temperature was set to 200 °C, while the mold temperature was maintained at 30 °C. The packing, holding, and filling pressures were all set to 0.4 MPa. The composite samples were fabricated using a continuous fiber 3D printing platform developed by our laboratory (Figure S16, Supporting Information). The process involved two main steps: i) applying an in situ coating of molten PLA/SSG particles onto flax fibers in the filament production line, and ii) feeding the prepared filament into a custom 3D printing system equipped with a flat nozzle for in situ shaping. During printing, the nozzle temperature was maintained at 200 °C, with a layer height of 0.35 mm, a line width of 0.12 mm, and a printing speed of 10 mm s^−1^.

### Characterization

The microstructures of the composites were examined by scanning electron microscope (SEM, Sigma 300, ZEISS, GER) and electronic video microscope (SANQTID, CHN). The interactions between PLA and SSG were analyzed using Fourier Transform Infrared Spectroscopy (FTIR, ISO 20, Nicolet, USA) with a scanning range of 400–4000 cm^−1^. The thermal stability of the blends was investigated using a Thermo Gravimetric Analyzer (TGA, Q500, TA, USA) with a temperature scanning interval of 30–600 °C and a ramp rate of 10 °C min^−1^. The dynamic mechanical responses of the PLA/SSG at various frequencies were analyzed using a Dynamic Mechanical Analyzer (DMA, Q800, TA, USA) with a three‐point bending mode, a clamp preload of 5 N, and a frequency scan range of 1– 50 Hz. The quasi‐tensile properties of PLA/SSG were measured with a Tinius Olsen universal testing machine (USA), following the specimen design guidelines outlined in GB/T 1040‐1BA. The impact energy absorption capabilities of PLA, PLA/SSG, and continuous fiber 3D printed composites were evaluated using a drop weight impact tester (CEAST 9350, Instron, USA). The dimensions of the impact specimens followed GBT229‐2020. The polymer specimens measured 80 mm × 10 mm × 4 mm, while the composite specimens were 80 mm × 10 mm × 8 mm. The effective span of the specimens was 60 mm, and both featured a notch‐free design (Figure S11, Supporting Information). All impact testing adhered to the relevant standards for impact bending tests,^[^ ^51^ ^]^ with an impact energy of 20 J for the PLA/SSG and 30 J for the PLA/SSG‐based continuous fiber‐reinforced composites.

## Conflict of Interest

The authors declare no conflict of interest.

## Supporting information



Supporting Information

Supplemental Video 1

## Data Availability

The data that support the findings of this study are available from the corresponding author upon reasonable request.
