# Supplementary material for: A Universal Toughening and Energy‐Dissipating Strategy for Impact‐Resistant 3D‐Printed Composites
Source: Adv Sci (Weinh). 2025 Apr 9;12(25):2501450. doi: 10.1002/advs.202501450 (PMC12224963; doi:10.1002/advs.202501450)
Supplement: Supplementary file 1 — Supporting Information [file ADVS-12-2501450-s002.docx]

Supporting Information

**A Universal Toughening and Energy-Dissipating Strategy for Impact-Resistant 3D-Printed Composites**

*Xiang Hong, Peng Wang*, Yu Ma, Weidong Yang, Junming Zhang, Zhongsen Zhang, Yan Li**

The fabrication process of PLA/SSG includes four main steps: (i) First, boric acid and hydroxyl silicone oil are thoroughly mixed in proportion and subjected to a polymerization reaction to obtain a polymer matrix with a silk-like luster (Figure S1). (ii) Next, oleic acid is added to the matrix for a plasticization reaction, producing SSG gel rich in dynamic "B-O" bonds, which will remain soft under slow compression while exhibiting a rapid increase in stiffness during fast compression (Figure S2). (iii) The SSG gel is then crushed into particles and blended with PLA in a molten state (Figure S3). The mixing ratios and sample numbers are detailed in Table S1 and Figure S4. (iv) Finally, PLA/SSG specimens are prepared using injection molding, and flax fiber-reinforced composites are fabricated using in-situ coating and continuous fiber 3D printing techniques (Figure S3).


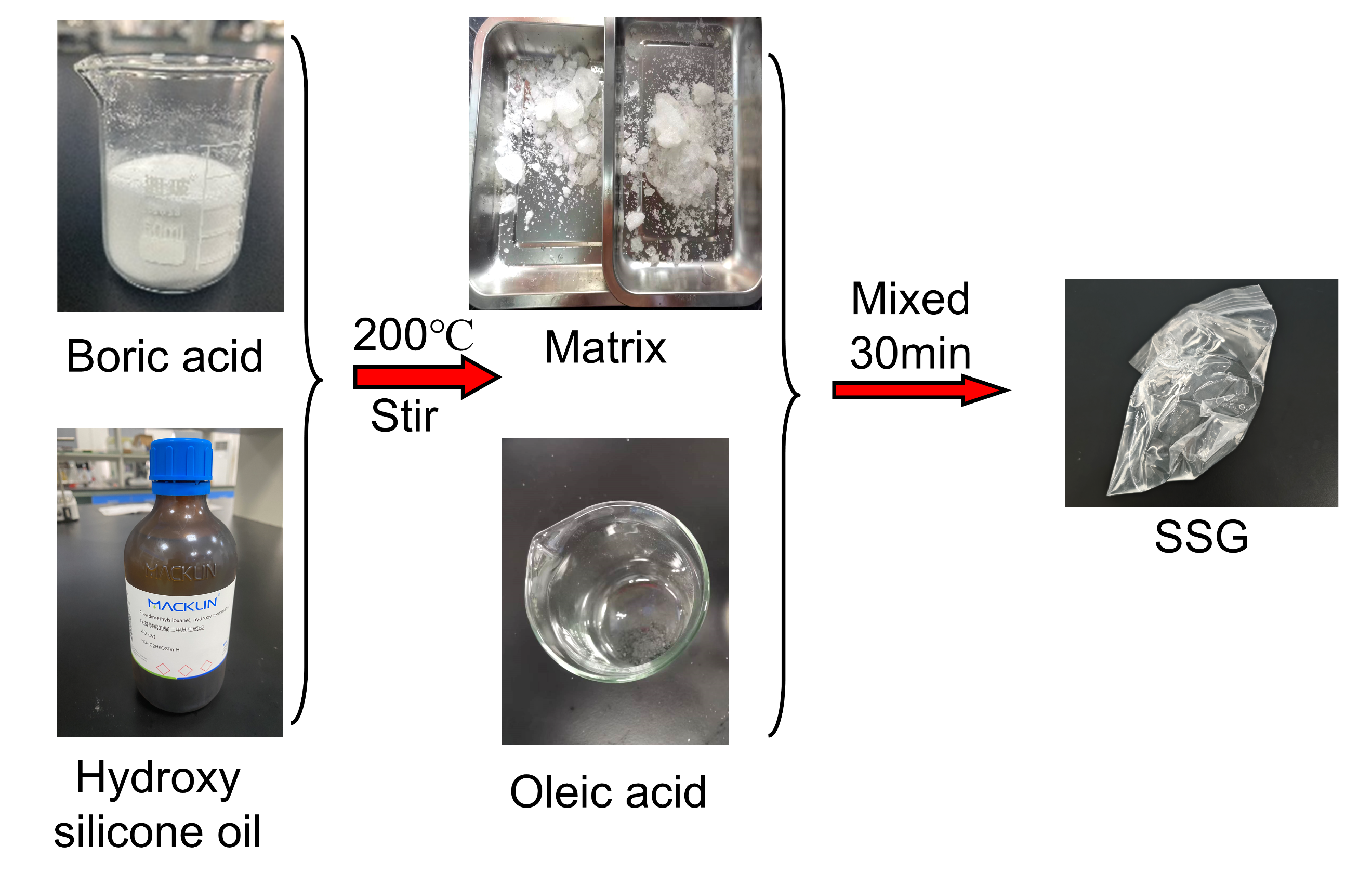


**Figure S1.** The preparation process of SSG and sample presentation (Boric acid, Hydroxyl silicone oil, and Oleic acid)


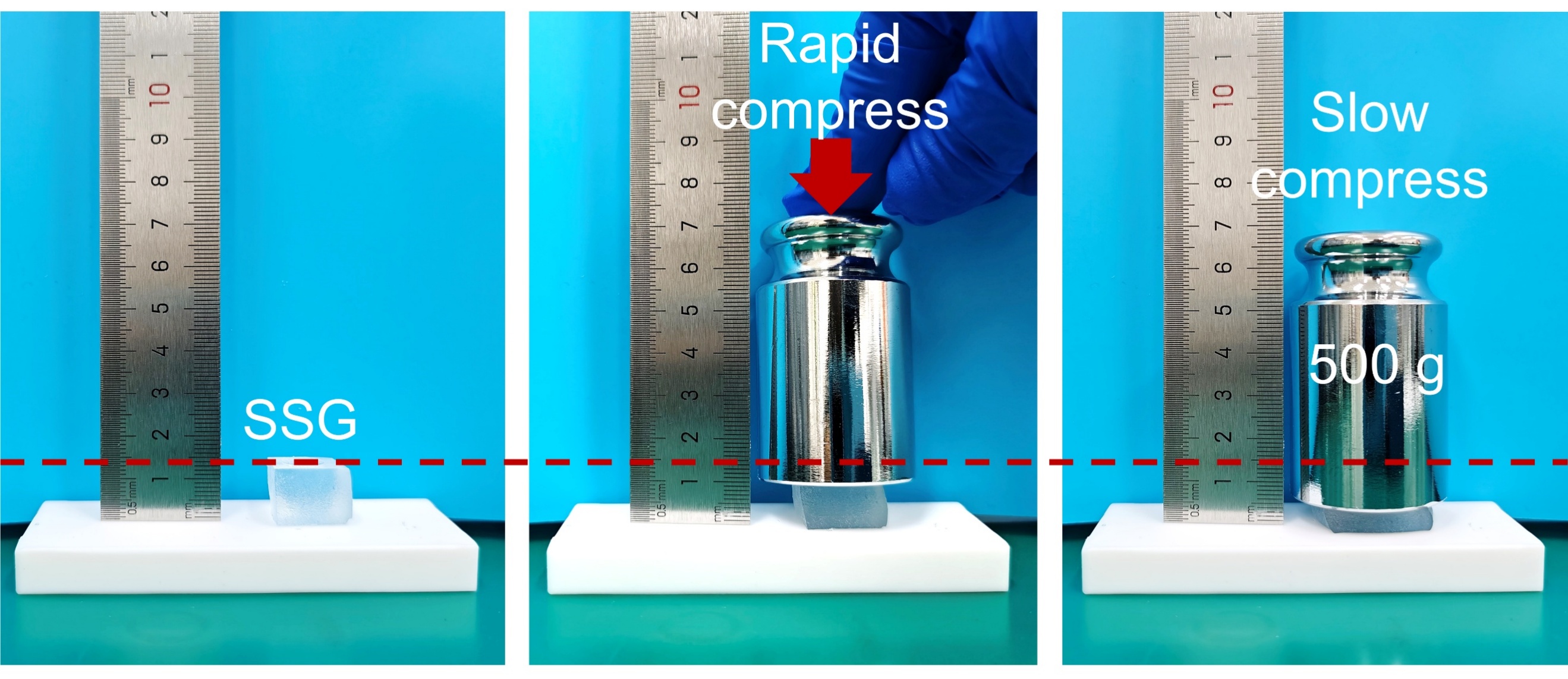


**Figure S2.** Shear-stiffening effect of SSG, the SSG remains soft under slow compression but exhibits a rapid increase in stiffness during fast compression.


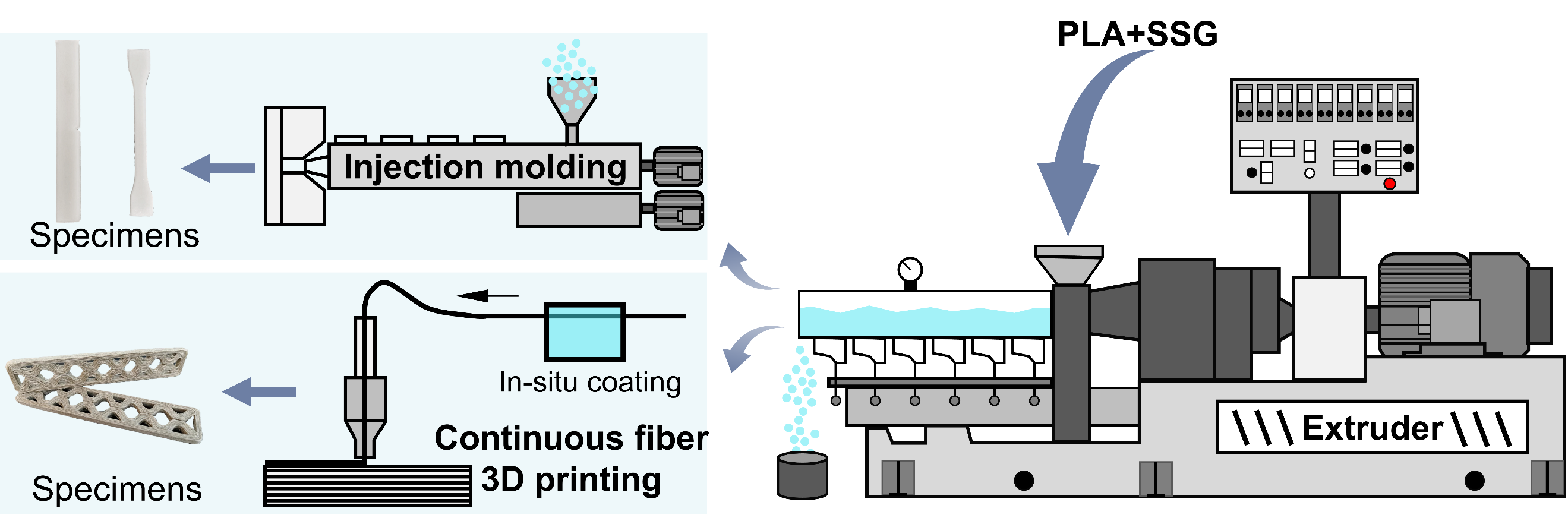


**Figure S3.** Melt blending, injection molding, and continuous fiber 3D printing process of PLA/SSG.


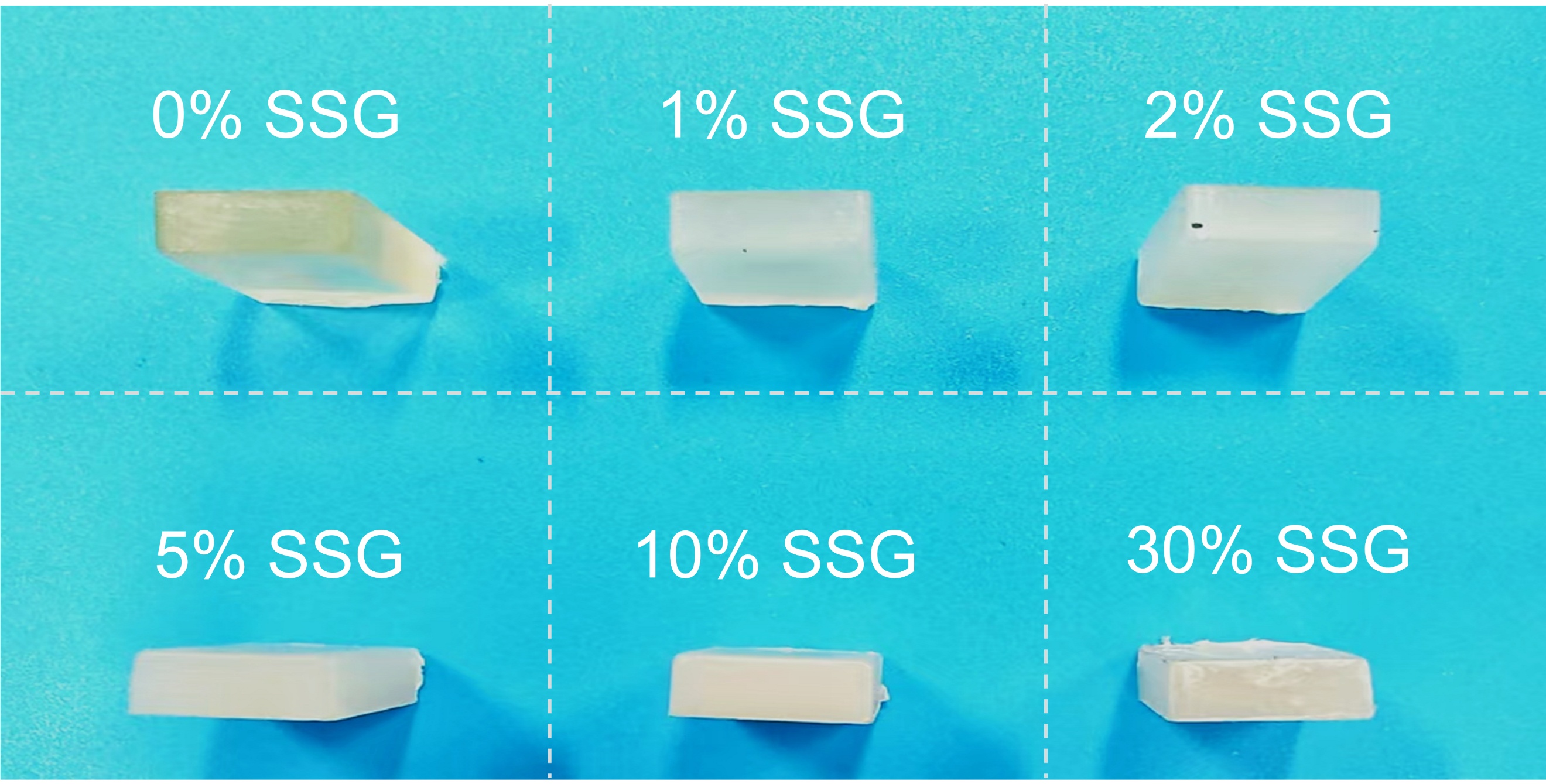


**Figure S4.** PLA/SSG composites with varying SSG content (As the SSG content increases, the color of the PLA/SSG mixture changes from transparent to pure white).

**Table S1** Weight percentages of the polymers used in the study

| **Types** | **PLA - 4032D** | **SSG** |
| --- | --- | --- |
| PLA | 100% | 0% |
| PLA/SSG (99/1) | 99% | 1% |
| PLA/SSG (98/2) | 98% | 2% |
| PLA/SSG (95/5) | 95% | 5% |
| PLA/SSG (90/10) | 90% | 10% |
| PLA/SSG (70/30) | 70% | 30% |
| SSG | 0% | 100% |

**Figure S5.** The chemical formula of SSG includes dynamic B-O bonds.


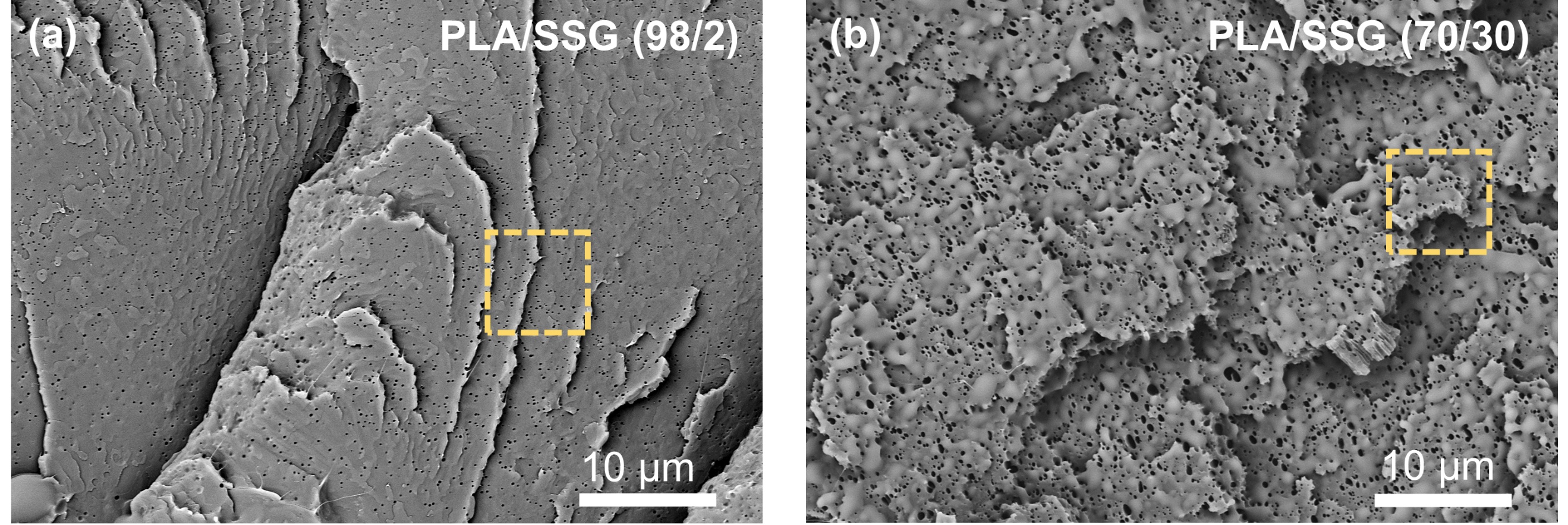


**Figure S6.** PLA/SSG composites with distinct river patterns morphology in the cross-section. a) PLA/SSG (98/2). b) PLA/SSG (70/30).

**Figure S7.** Particle size distribution of SSG in the composites. a) PLA/SSG (98/2). b) PLA/SSG (70/30).

**Figure S8.** The typical tensile stress-strain curve of PLA/SSG consists of four stages: (1) Elastic stage, the stress and strain follow a linear relationship governed by Hooke’s Law, deformation primarily results from changes in bond length and angle, which are both reversible and uniform. The slope of this stage represents Young’s modulus. (2) Necking stage, the stress reaches yield strength, leading to plastic deformation of the material. After the yield point, a phenomenon called "strain softening" takes place, leading to the formation of a local "neck" in the sample, where molecular chain segments align in the direction of the applied tensile force. (3) Plastic deformation stage, as the neck gradually expands, the material undergoes significant plastic deformation. In addition, the oriented molecular chains resist further stretching, and multiple cracks induced by stress concentration in SSG hinder the propagation of macroscopic cracks, causing a slight increase in stress until final fracture occurs.


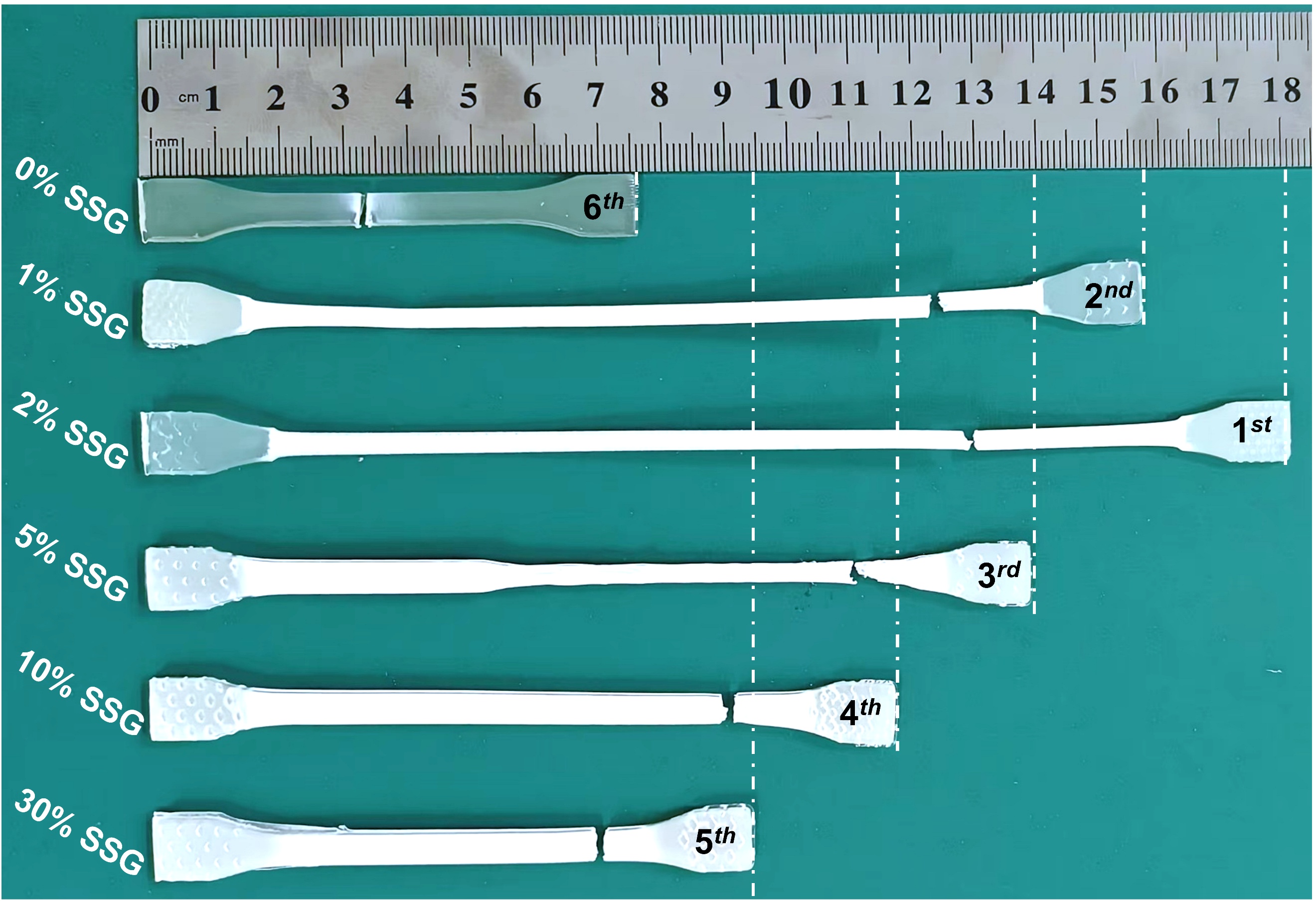


**Figure S9.** Macroscopic fracture morphology of various types of PLA/SSG after static tensile loading.


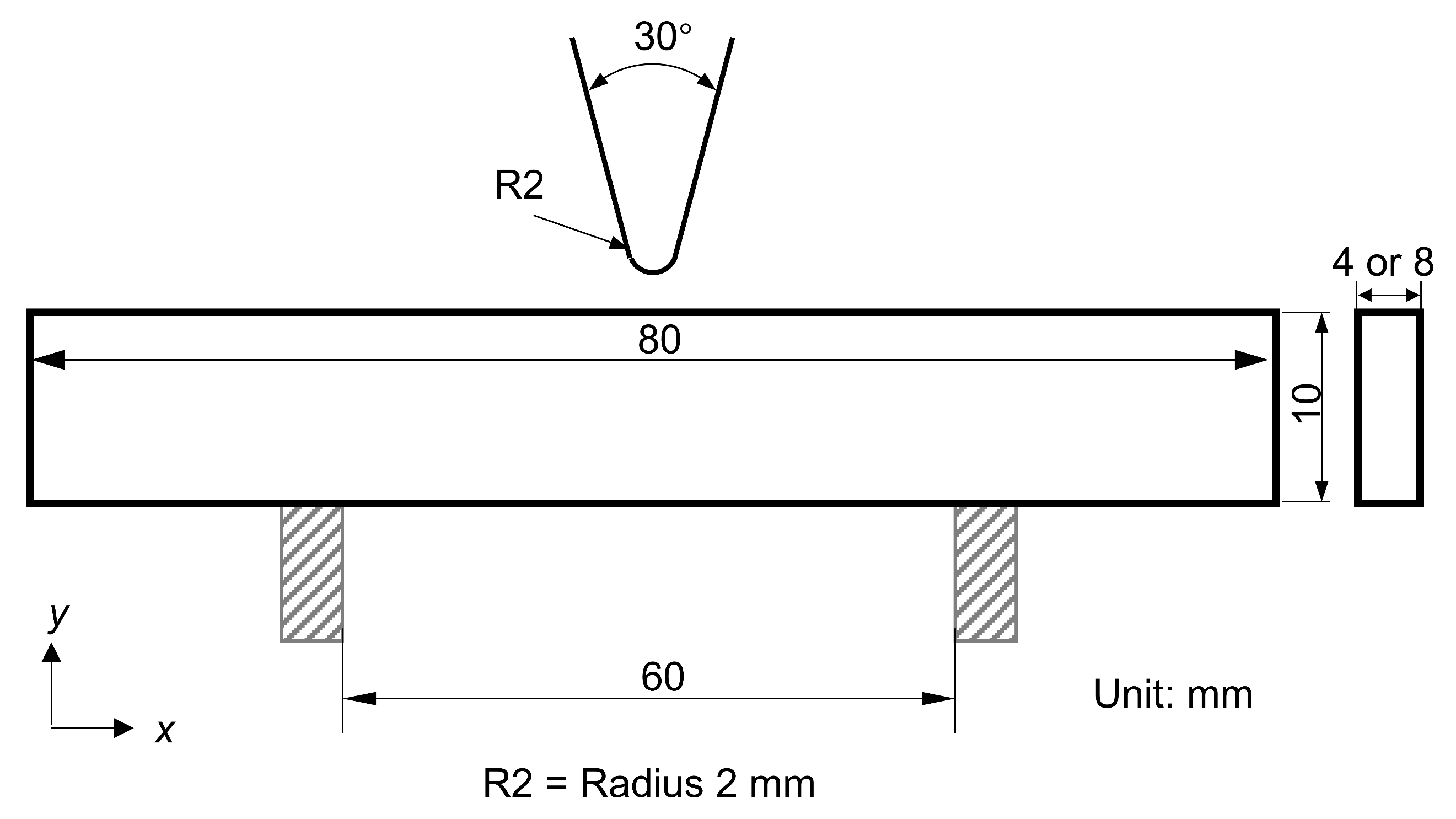


**Figure S10.** Schematic of the impact bending specimen (For PLA/SSG: the dimension is 80 mm × 10 mm × 4 mm; For PLA/SSG-based continuous fiber reinforced composite: the dimension is 80 mm × 10 mm × 8 mm).


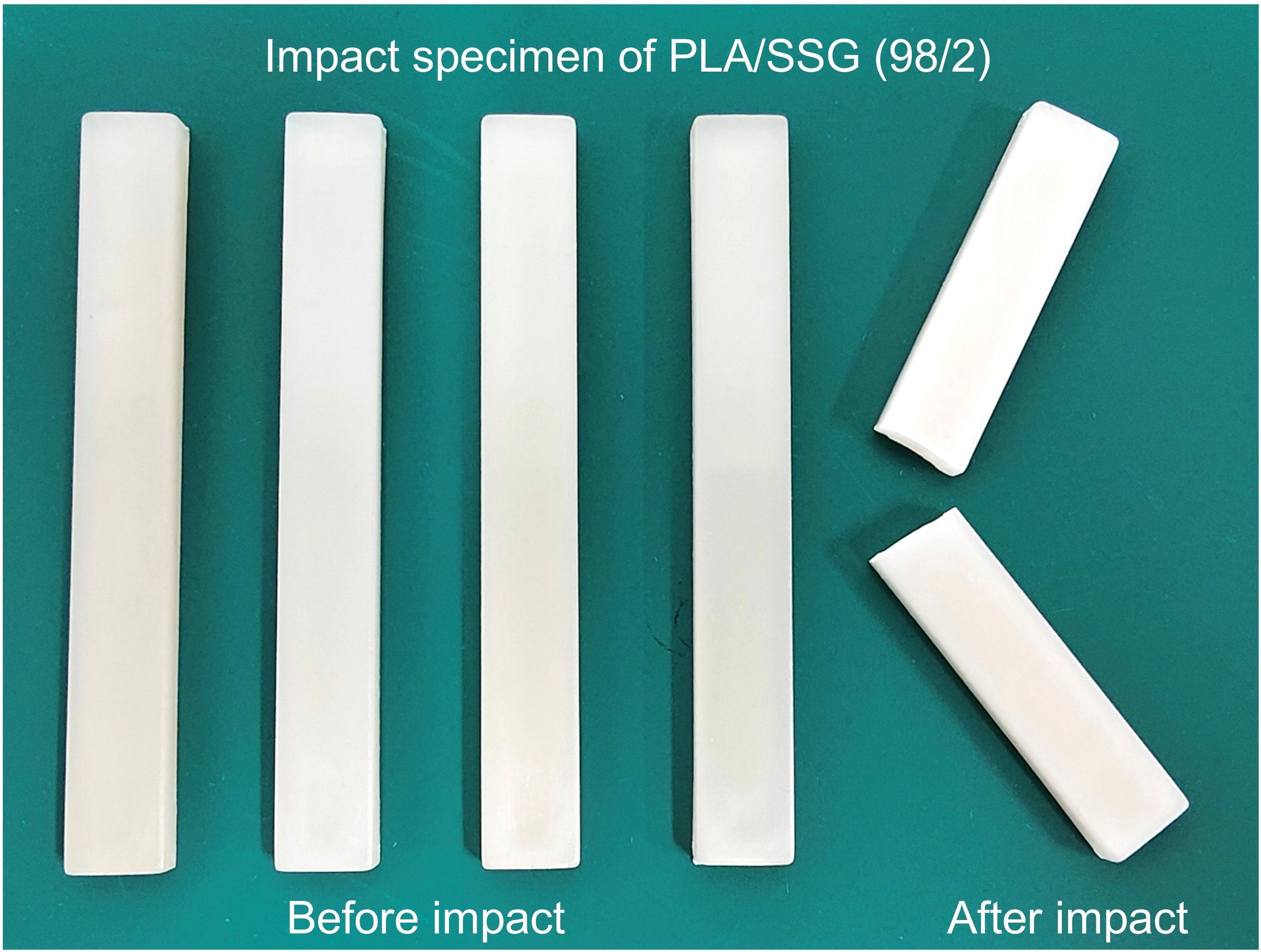


**Figure S11**. PLA/SSG samples before and after impact bending tests, with a minimum of five samples in each experimental group.


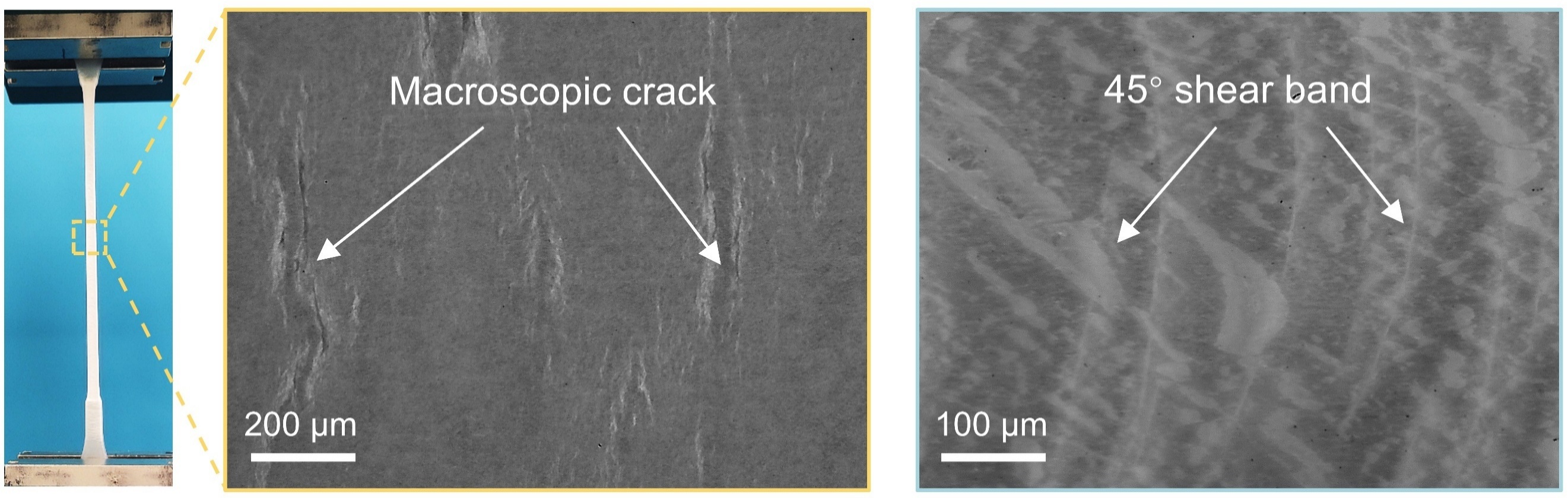


**Figure S12.** The 45° shear band and macroscopic crack phenomena of PLA/SSG during loading.


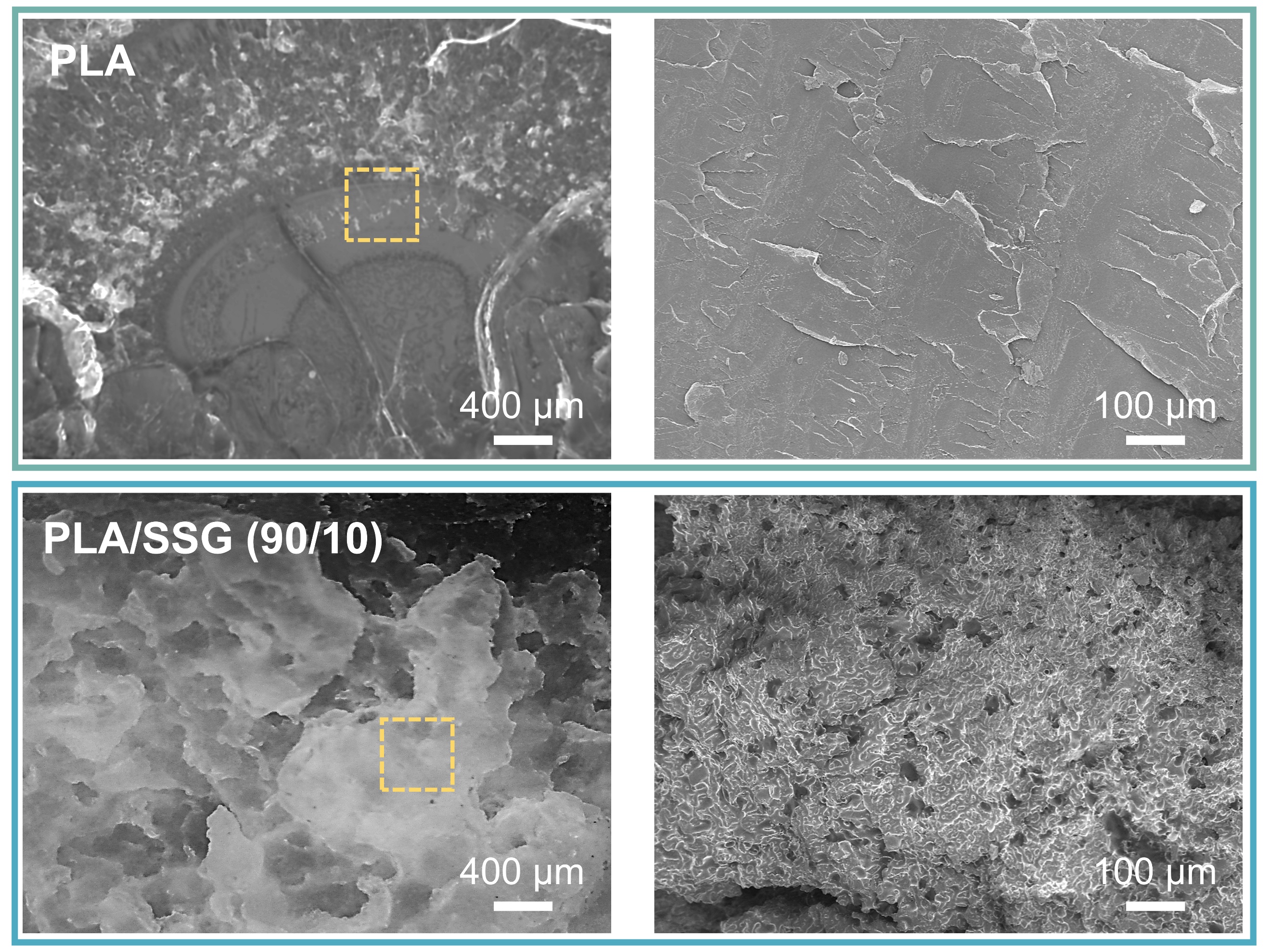


**Figure S13.** Cross-sectional morphology of PLA and PLA/SSG (90/10) after impact at various magnifications


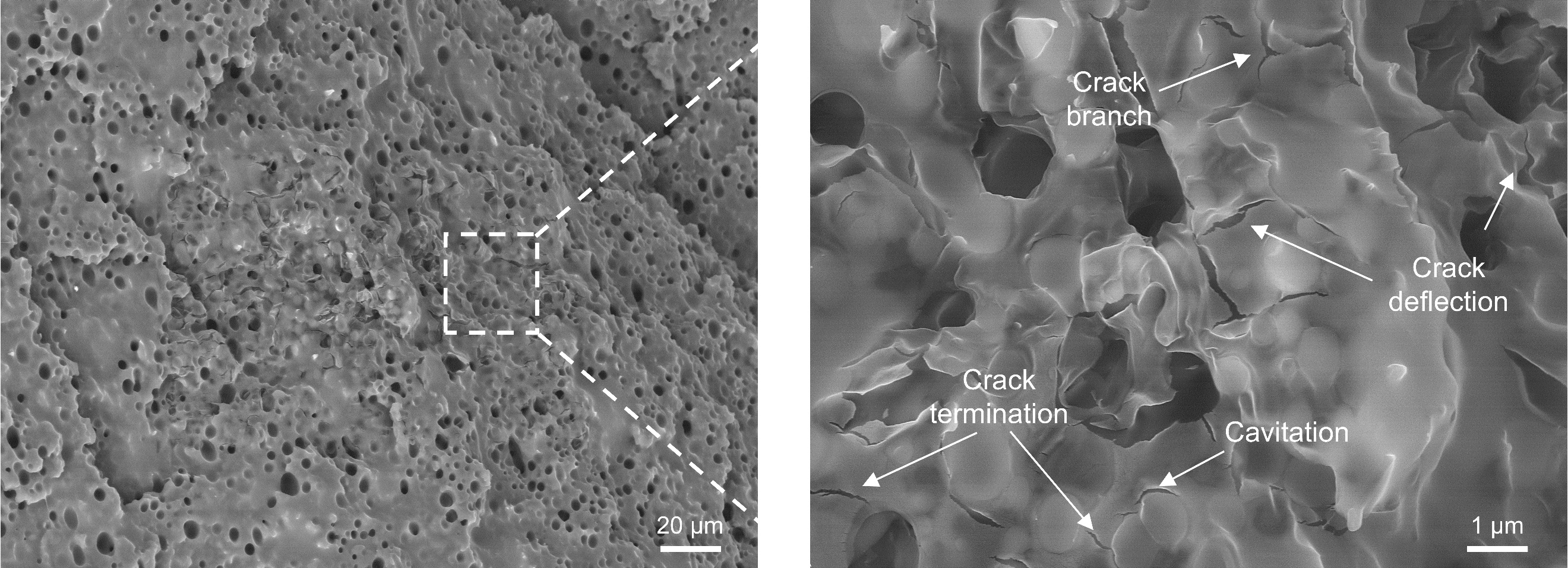


**Figure S14.** The fracture cross-section of PLA/SSG after tensile testing, exhibiting cavitation debonding, crack deflection, branching, and termination.


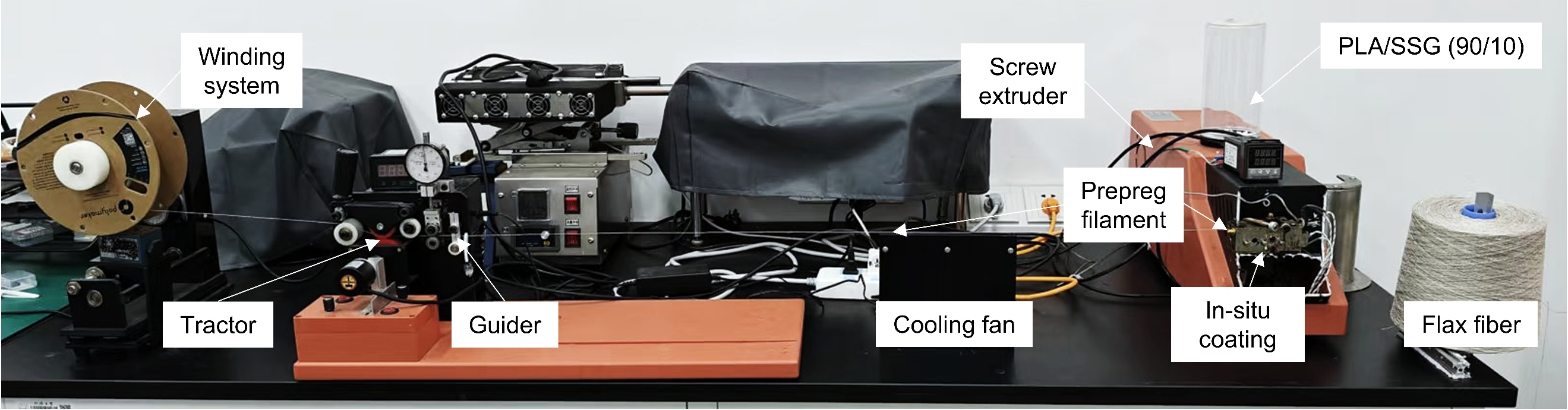


**Figure S15.** The *in-situ* coating process of flax fiber based on PLA/SSG (90/10), including 4 key steps: (1) Initially, the flax fiber yarn is unwound from the system. (2) The fiber then passes through a customized impregnation unit, where it undergoes multi-stage roller pressing in a 200°C molten resin bath to ensure thorough impregnation of the fiber bundles. (3) Next, the fiber is cooled and set through a gradient cooling module, which solidifies the PLA/SSG coating. (4) Finally, the trajectory of the impregnated filament is adjusted by a path-guiding wheel, and it is wound into shape by a tension-controlled winding system set to a pulling speed of 2000 mm/min. This results in the production of continuous flax fiber reinforced prepreg filament suitable for 3D printing.


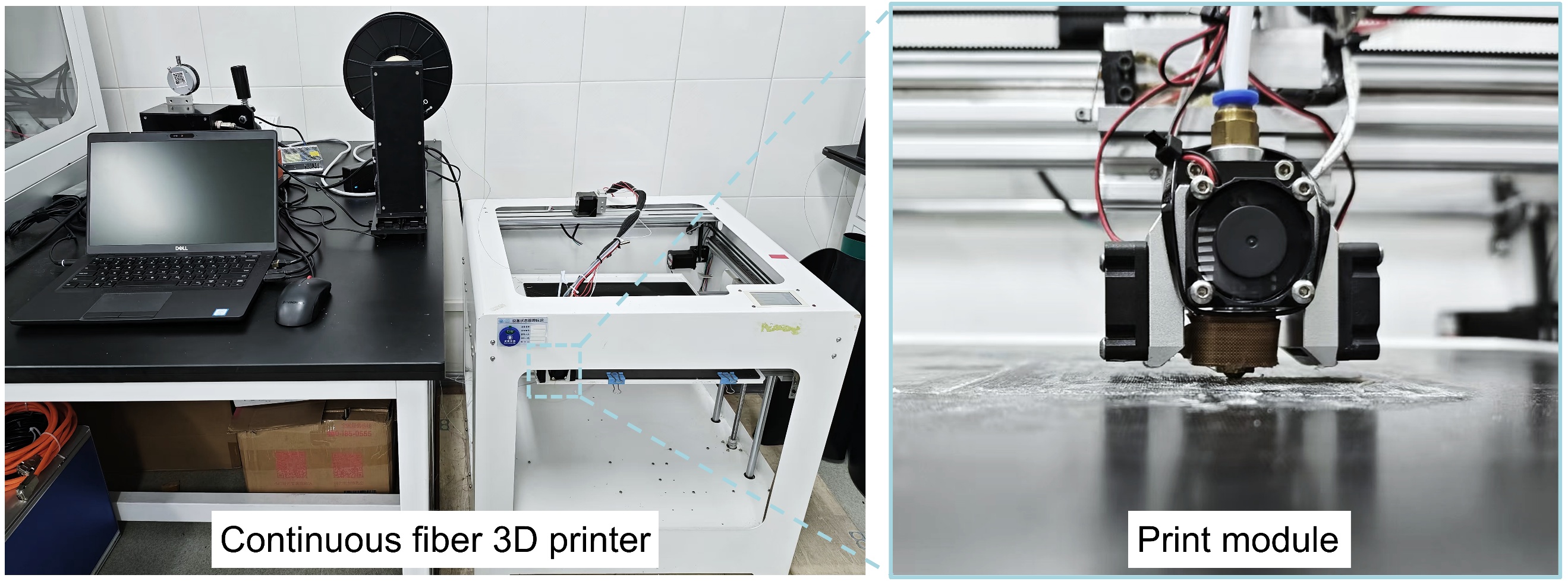


**Figure S16.** Continuous fiber 3D printer and enlarged view of the print module.
